# Embedded Vision Intelligence for the Safety of Smart Cities

**DOI:** 10.3390/jimaging8120326

**Published:** 2022-12-14

**Authors:** Jon Martin, David Cantero, Maite González, Andrea Cabrera, Mikel Larrañaga, Evangelos Maltezos, Panagiotis Lioupis, Dimitris Kosyvas, Lazaros Karagiannidis, Eleftherios Ouzounoglou, Angelos Amditis

**Affiliations:** 1 Fundación Tekniker, 20600 Eibar, Spain; 2 Institute of Communication and Computer Systems (ICCS), 15773 Zografou, Greece

**Keywords:** smart cities, edge, EdgeX Foundry, embedded machine vision, artificial intelligence, deep learning

## Abstract

Advances in Artificial intelligence (AI) and embedded systems have resulted on a recent increase in use of image processing applications for smart cities’ safety. This enables a cost-adequate scale of automated video surveillance, increasing the data available and releasing human intervention. At the same time, although deep learning is a very intensive task in terms of computing resources, hardware and software improvements have emerged, allowing embedded systems to implement sophisticated machine learning algorithms at the edge. Additionally, new lightweight open-source middleware for constrained resource devices, such as EdgeX Foundry, have appeared to facilitate the collection and processing of data at sensor level, with communication capabilities to exchange data with a cloud enterprise application. The objective of this work is to show and describe the development of two Edge Smart Camera Systems for safety of Smart cities within S4AllCities H2020 project. Hence, the work presents hardware and software modules developed within the project, including a custom hardware platform specifically developed for the deployment of deep learning models based on the I.MX8 Plus from NXP, which considerably reduces processing and inference times; a custom Video Analytics Edge Computing (VAEC) system deployed on a commercial NVIDIA Jetson TX2 platform, which provides high level results on person detection processes; and an edge computing framework for the management of those two edge devices, namely Distributed Edge Computing framework, DECIoT. To verify the utility and functionality of the systems, extended experiments were performed. The results highlight their potential to provide enhanced situational awareness and demonstrate the suitability for edge machine vision applications for safety in smart cities.

## 1. Introduction

Ensuring citizens’ safety and security has been identified as the number one priority for city authorities when it comes to the use of smart city technologies. Automatic understanding of the scene, and the associated provision of situational awareness for emergency situations and civil protection, are able to efficiently contribute to such domains. Traditional video surveillance systems demand human intervention to some extent. However, as the number of IP or other types of cameras increases explosively, a fully automatic video recognition framework becomes essential, replacing the manual monitoring. The video data captured by the camera are transmitted to the cloud server to do the entire recognition process, which may hamper real-time video recognition due to transmission delays through the communication channel. In this context, recent trends in Internet of Things (IoT) applications adopt edge computing that appears to decrease latency and computational processing. The edge computing technology allows computation to be performed at the network edge so that computing happens near data sources or directly in the real-world application as an end device [1]. In these terms, edge computing could scale from a single person to a smart individual building to even an entire city.

This is possible due to advances in the manufacturing of new processors [2]. Such devices request services and information from the cloud, as well as perform several real-time computing tasks (e.g., storage, caching, filtering, processing, etc.) of the data sent to and from the cloud. Although in [3] the authors highlighted some drawbacks and aspects that should be considered when applying edge computing such cases of unreliable devices, possible low computing power of individual edge notes, load balancing, high operational expenses, and concerns in the system’s security and trustworthiness [4,5,6], edge computing is able to fully contribute to situational awareness applications [7,8]. Situational awareness applications continuously generate enormous amounts of data of several types. Edge computing is able to provide a homogeneous approach for data processing and generation of associated alerts or events or raw information. In summary, edge computing can be used for real-time smart city environments under the public safety aspect, enabling (i) context-awareness, (ii) geo-distributed capabilities, (iii) low latency, and (iv) migration of computing resources from the remote cloud to the network edge.

In this background, [9] provided in their study a modular architecture with deep neural networks as a solution for real-time video analytics in an edge-computing environment. In their modular architecture, two networks of Front-CNN (Convolutional Neural Network) and Back-CNN were exploited. Experimental results on public datasets highlighted the potential of their approach. In [10], a video streaming optimization method in the IIoT environment was proposed under the edge computing concept. In the same framework, the author of [11] designed an edge enhanced deep learning system for large-scale video stream analytics system. In their proposed methodology, they performed an initial processing of the data close to the data source at edge and fog nodes, resulting in significant reduction in the data that are transferred and stored in the cloud. The results on the adopted object recognition scenario showed high efficiency gain in the throughput of the system by employing a combination of edge, in-transit and cloud resources when compared to a cloud-only approach. The authors of [12] focused on leveraging edge intelligence for video analytics in smart city applications. Their approach encompasses architecture, methods, and algorithms for (i) dividing the burdensome processing of large-scale video streams into various machine learning tasks, and (ii) deploying these tasks as a workflow of data processing in edge devices equipped with hardware accelerators for neural networks. In [13], the authors investigated an architectural approach for supporting scalable real-time video stream processing using edge and in-transit computing. Concerning the privacy aspect, the authors of [14] proposed how to consider to a privacy-oriented framework when video feeds are exploited for surveillance applications.

Other recent works emphasized the important role of edge computing for several sectors, such as automated manufacturing [15], healthcare systems and Industry 4.0 [16], telecommunications [17], autonomous traffic systems [18] and smart cities [19,20,21,22]. Through the continuous advancements of ubiquitous computing, wireless sensor networks and machine-to-machine communication (M2M), connected devices are growing in number. Heterogeneous physical devices are enabled to transfer signals over the internet and become uniquely identifiable [20]. A sustainability roadmap for edge computing was proposed in [23]. Their approach aims to serve the developers’ and managers’ communities in the edge computing field to include sustainability dimensions, such as the usage of distributed renewable energy sources for edge nodes, as concerns that are as important as the technical concerns ensuring the functionality and efficiency of edge computing. Edge computing has been also identified as an enabling technology for numerous use cases in the development of Internet of Things and the 5th Generation Network (5G) [24] and is, therefore, an important piece to study. In [25], a cyber-physical social system for early fire detection was proposed, employing a scalable edge computing framework receiving captured information from several IoT nodes. In [26], the authors developed a fire alarm system for smart cities using edge computing via different types of sensors, such as temperature and humidity; whenever a node detects a fire, it signals the centralized node to alert the user.

Finally, with the complex structures of hierarchical layers being able to capture features from raw data, deep learning models have shown outstanding performances in several novel applications, such as machine translation, object detection, smart question and answer systems, and semantic segmentation [27,28,29]. Traditionally, most deep learning-based applications are deployed on a remote cloud center, and many systems and tools are designed to run deep learning models efficiently on the cloud. Recently, with the rapid development of edge computing, the deep learning functions are being offloaded to the edge. Thus, it calls for new techniques to support the deep learning models at the edge [30]. In this context, many deep learning packages have been widely used to deliver the deep learning algorithms and deployed on the cloud data centers, including TensorFlow [31], Caffe [32], PyTorch [33], and MXNet [34]. However, the models deployed in the cloud are usually computationally expensive and traditionally executed in a high-end computer equipped with powerful and expensive graphical processing units (GPU). This equipment is not adequate for a large-scale deployment because of the high power-consumption, the lack of miniaturization and the resultant high costs. To solve this issue and motivated by the great success of the deep learning techniques, many hardware manufactures have developed embedded hardware devices powerful enough to execute some of the most successful models. In addition, those devices usually integrate a powerful co-processor specifically designed for the deployment of deep learning models. These co-processors provide considerable computing power along with small footprint for high integration and high-power efficiency, and support new edge-based deep learning frameworks, tools and lightweight solutions, such TensorFlow lite [35], Caffe2 [36], DeepViewRT [37], or ONNX [38].

The work in this paper has been supported within the S4AllCities EU funded project [39]. The S4AllCities project aim is to make cities’ infrastructures, services, ICT systems and Internet of Things more resilient, while promoting intelligence and information sharing amongst security stakeholders. The objective of this work is to highlight the utility and functionality of two different schemes of embedded vision systems with edge computing capabilities in smart city applications focused on person detection in terms of situational awareness. Furthermore, their overview and the performance results via extended experiments in real scenes are provided. Both edge computing machine vision systems run deep learning models to detect people and suspicious behaviors. These detections are notified as events to safety forces through an edge device scheme based on the EdgeX Foundry framework [40] to increase the safety on the future smart cities.

### 1.1. Edge Computing Frameworks

The authors of [30] listed several edge computing systems, projects and tools that were designed and developed in previous years for various applications (general usage scenario, vehicular data analytics, smart home, video stream analytics, virtual reality), such as: (i) Cloudlet [41], (ii) CloudPath [42], (iii) PCloud [43], (iv) ParaDrop [44], (v) SpanEdge project [45], (vi) Cloud-Sea Computing Systems project [46], (vii) Cachier [47], (viii) Precog [48], (ix) FocusStack [49], (x) AirBox [50] and (xi) Firework [51]. On the other hand, several open-source edge computing frameworks have gained significant attention over the last years due to their open use, efficiency and flexibility. Some well-known open-source edge computing frameworks are [30] (i) CORD (Central Office Re-architected as a Datacenter) [52], (ii) Akraino Edge Stack [53], (iii) EdgeX Foundry [40] and (iv) Apache Edgent [40,54].

The authors of [30] summarized the features of the above open-source edge computing systems and compared them from different aspects, including the main purpose of the systems, application area, deployment, target user, virtualization technology, system characteristic, limitations, scalability and mobility. According to such a comparison, as well as recent studies [55,56,57,58], the EdgeX Foundry is considered in the bibliography as a highly flexible and scalable edge computing framework facilitating the interoperability among devices and applications at the IoT edge, such as industries, laboratories, and datacenters. Hosted on a reference software platform that is completely independent of hardware and operating systems, the EdgeX Foundry framework enables a plug-and-play component ecosystem to unify the computing open platform at the edge of the IoT and accelerate deployment of solutions [55]. It is also considered as a lightweight edge computing framework [56] and supports the connections to heterogeneous device protocols, providing various management functions for devices, data, and edge computing environments. Additionally, according to several organizations (e.g., Alliance for Internet of Things Innovation-AIOTI [59]), it is recognized, among others, as one of the Open-Source Software (OSS) initiatives that are currently focusing on edge computing.

### 1.2. Low-Cost Edge Platform with a Dedicated DL Co-Processor

There are many hardware devices competent to execute DL algorithms. In fact, most modern micro-controllers are currently able to run a set of DL algorithms [55]. However, one of the goals of this work is to deploy machine vision DL algorithms with high computing power demand. On average, the number of operations required to compute a complete inference from an input image is around some tens of billions of operations or Giga-Operations (GOPS) [60]. Considering a video sequence of 30 to 60 frames per second, it is estimated that the minimum computational power is around one Tera-Operations per second (TOPS). That figure leads to the consideration of a device with a specific DL integrated mathematical co-processor.

There are three main solutions to integrate a DL-oriented co-processor in embedded hardware:

1. Use a general-purpose processor that already integrates a co-processor in the same semiconductor die. This solution provides high integration, high speed inter-processor communication and simplified hardware design, but it is less versatile and adaptative to changes in application requirements.
2. Include a separate Application Specific Integrated Circuit (ASIC) designed for DL inference, together with the general-purpose processor in the embedded hardware design, allowing the separation of the general-purpose processing and peripheral handling and the DL processing issues. In exchange, the chipset intercommunication and synchronization should be solved.
3. Use a programmable logic device (CPLD or FPGA) to implement custom co-processor hardware. This is the most versatile alternative that even allows the design of the architecture of the DL co-processor itself but involves more complex development and design stages.

There are many examples of those solutions in the market. The survey in [61] describes some of most successful embedded AI application development platforms. The Nvidia Jetson Nano [62], for example, uses an embedded floating point Graphical Processing Unit (GPU) with a highly parallel architecture of 128 cores to run deep learning applications. The evaluation kit specifies a 472 Giga Floating-Point Operations per Second (GFLOPS) of computing power. Another very popular device is Google’s EdgeTPU coprocessor [63]. The device is an ASIC designed to specifically run Neural Network inference at the edge with up to 4 Tera operations per second (TOPS) of computing power. The EdgeTPU, as opposed to Jetson Nano, performs the mathematical operations using 8-bit fixed point integer arithmetic. This feature allows very high inference speed but at limited accuracy and, as it is explained below, the architecture has a notable impact in the software deployment stage. A representative example of an FPGA based AI development board is the Ultra96 board based on a Xilinx System on Chip (SoC) which integrates an FPGA fabric and a quad ARM Cortex A54 cores [64]. This mixed architecture could be scaled to a high-performance AI-oriented-design-based FPGA with even hundreds of TOPS of computing power such as, for example, the Intel Stratix 10 NX FPGA [65]. However, the implementation of a specific IP core for machine learning model execution presents some challenges that should be carefully considered [66].

## 2. Materials and Methods

This section presents the hardware and software modules developed for Edge computing machine vision purposes in the context of the S4AllCities project. First, the Distributed Edge Computing framework (DECIoT), an edge computing framework for the management of edge devices, is introduced. Then, a custom hardware platform specifically developed for the deployment of deep learning models is presented and a people detection application is integrated with the DECIoT. Finally, a custom Video Analytics Edge Computing (VAEC) system is deployed on a commercial NVIDIA Jetson TX2 platform and integrated with the DECIoT, providing enhanced situation awareness for person detection.

### 2.1. Distributed Edge Computing Framework (DECIoT) for the integration of Edge Devices

The designed and developed DECIoT platform for the S4AllCities project is based on the EdgeX Foundry. In general, the DECIoT platform is able to address, among others, the problem of gathering, filtering and aggregating data, it allows the interoperability between devices, interacts with the IoT devices, provides security and system management, provides alerts and notifications, executes commands, stores data temporarily for local persistence, transforms/process data and, in the end, exports the data in formats and structures that meet the needs of other platforms. This whole process is performed by using open-source microservices that are state-of-the-art in the area of distributed edge IoT solutions. The DECIoT is a scalable, secure, flexible, fully controlled, potentially interoperable, and modular open-source framework that ensures information sharing with other platforms or systems. Through the DECIoT, computation, data storage, and information sharing are performed together, directly in the edge device, in a real-time manner.

**Figure 1:**
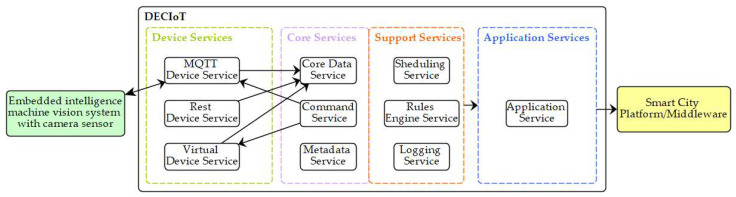
DECIoT architecture. The four main different layers of the proposed edge computing framework. From left to right: (i) the Device Service Layer acts as an interface of the system with physical devices and is tasked with the functionality of collecting data and actuating the devices with command; (ii) the Core Services Layer is used for storing data, commanding and registering devices; (iii) the Support Services Layer includes microservices for local/edge analytics and typical application duties, such as logging, scheduling, and data filtering; (iv) the Application Services Layer consists of one or more microservices to extract, transform, and send data from the previous layer to other endpoints or applications.

The DECIoT platform consists of multiple layers and each layer contains multiple microservices. The communications between the micro-service within the same or different layers can be performed either directly with the use of REST APIs or with the use of a message bus that follows a pub/sub mechanism. Both schemes are exploited in this study. DECIoT consists of a collection of reference implementation services and SDK tools. The micro services and SDKs are written in Go [67] or C programming languages. A detailed documentation and implementation of the DECIoT is provided in [68,69]. In the following, we present the main different layers of DECIoT (see also Figure 1):

- The Device Service Layer: Acts as an interface of the system with physical devices and is tasked with the functionality of collecting data and actuating the devices with commands. It supports multiple protocols for communication though a set of device services (MQTT Device Service, REST Device Service, Virtual Device Service, etc.) and an SDK for creating new Device Services. Here, the MQTT Device Service was used to receive information from the person detection process. Between the person detection process and the MQTT Device Service, there is a MQTT broker (Mosquito) [70].
- The Core Services Layer: Located at the center of the DECIoT platform and is used for storing data, as well as commanding and registering devices. The Core Data Service is used for storing data, the Command Service initiates all the actuating commands to devices, and the Metadata Service stores all the details for the registered devices. This microservices are implemented with the use of Consul, Redis, and adapters developed in Go for integration with all other microservices.
- The Support Services Layer: Includes microservices for local/edge analytics and typical application duties, such as logging, scheduling, and data filtering. The Scheduling Service is a microservice capable of running periodic tasks within DECIoT (for example, cleaning the database of the Core Data Service each day) and initiating periodic actuation commands to devices using the Command Core Service. This is an implementation in Go that exploit features of Consul and Redis. The Rules Engine Service performs data filtering and basic edge data analytics, and Kuiper is used in this microservice. The Logging Service, a Go language implementation, is used for logging messages of other microservices. Here, the relevant microservices were not exploited as no logging, scheduling, and data filtering was needed.
- The Application Services Layer: Consists of one or more microservices with the functionality of communicating with external infrastructures and applications. Application Services are the means to extract, transform, and send data from the DECIoT to other endpoints or Applications. Using this Layer, the DECIoT can communicate with a variety of middleware brokers (MQTT, Apache Kafka [71], etc.) or REST APIs with the goal of reaching external applications and infrastructures. At the same time, the support of an SDK allows the implementation of new Application Services that fit the use case. Here, a new Application Service has been implemented to send data to the smart city’s middleware (in this study, the Apache Kafka was used) using the Go language.

**Figure 2:**
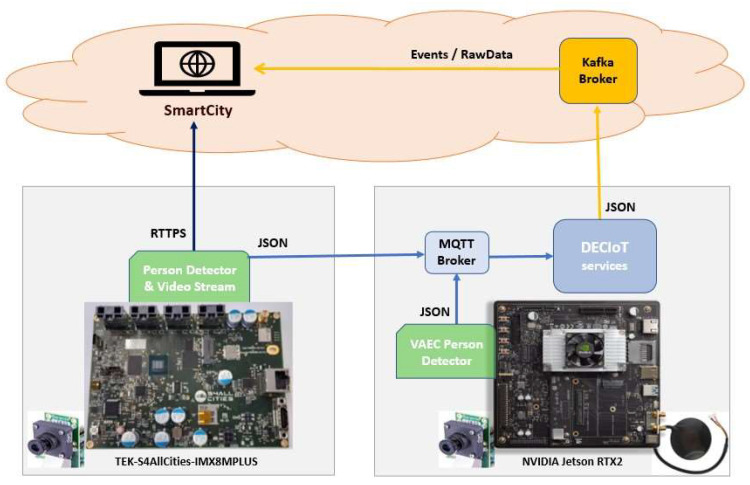
S4AllCities schema with ICCS (VAEC via NVIDIA Jetson RTX2) and Tekniker (I.MX8M Plus) edge platforms transmitting video streaming and the number of people detected.

In the context of S4AllCities project, the proposed DECIoT framework is used to integrate embedded edge processing platforms with the Smart City Middleware (Figure 2). On the one hand, a low-cost custom platform based on the NXP I.MX8M Plus SoC is presented. This platform makes use of novel hardware specific tools, such as the NXP Image Signal Processing (ISP) and the DeepViewRT model inferencing framework, to reduce the processing time of a person detector application. In case of necessity, the image can be streamed to the smart city for manual human-recognition purposes. On the other hand, a commercial NVIDIA Jetson RTX2 is used to deploy and evaluate a pre-trained and a custom YOLOv5s based model, able to detect people in crowded indoor or outdoor scenarios and from different terrestrial or aerial perspectives. Being a more powerful platform, the NVIDIA Jetson RTX2 platform has been also used as a host server for the deployment of the DECIoT services. These services have the capability for: (i) collecting the alerts or raw data generated by edge devices through different IoT communication protocols; (ii) post-processing the information from different sources, taking into account historical data to detect specific events and generate the corresponding alerts; (iii) filtering and sending the most interesting events to higher-level layers using different communication protocols.

This work also presents two scenarios where the edge devices send the number of people detected to the DECIoT through the MQTT protocol. The DECIoT framework processes the incoming data and, using the communication microservices based on Kafka, transmit events to the smart city platform.

### 2.2. AI Embedded Platform

**Figure 3:**
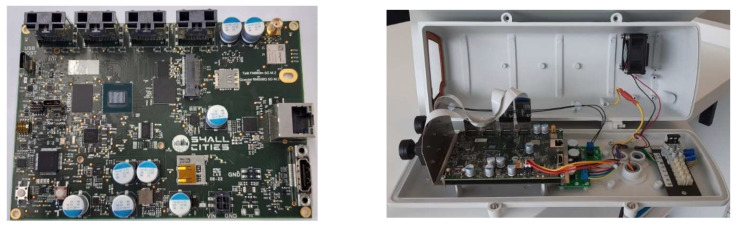
S4AllCities Hardware platform based on the I.MX8M PLUS and enclosed as a security inspection camera.

Based on the criteria presented in Section 1.2, the AI hardware selected for Tekniker AI Embedded Platform was the I.MX8M Plus processor. It is an NXP heterogeneous multi-core processor for high-performance applications focused on video processing and DL [72]. Based on this processor, the edge processing machine vision device was developed and manufactured (Figure 3).

The I.MX8M Plus processor for machine vision integrates a Neural Co-Processing Unit (NPU) which can process 2.3 Tera-operations per second (TOPS), enabling the NPU to run deep learning model inference in nearly real time. It is also quite remarkable that the NPU is integrated onto the same die as the general-purpose processors and shares the high-speed internal memory bus. This architecture helps speed up the Deep Neural Network (DNN) based inference since the data interchange between both computing units is optimized. The NPU is a Vivante VIP8000 specifically designed for being embedded in processors of the i.MX family. It works with 8-bit integer data types (INT8) rather than 32-bit floating-point data (FLOAT32). This means that the DNN needs to be transformed (quantized) before being executed in the NPU. NXP provides the entire ecosystem of tools to manage the entire workflow pipeline, including the design, deployment and inference of neural networks. The processor also features a powerful image-processing pipeline, camera interfaces and a comprehensive set of communication peripherals.

The embedded software is based on a Linux distribution created using Yocto project. The Yocto framework allows creating and configuring a custom Linux distribution with the minimum packages, libraries and drivers that exactly fit the application requirements based on the hardware devices and use case functionalities. The Yocto version used was Yocto Zeus (v5.4.70_2.3.2) (Variscite, Lod, Israel).

#### 2.2.1. NXP Enhanced Solutions to Accelerate Machine Learning Application’s Performance

**Figure 4:**
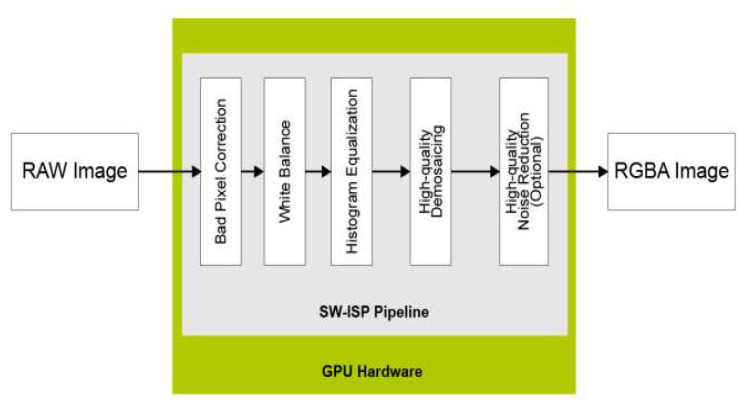
NXP Image Signal Processing Software (optimized by OpenCL 1.2 and OpenVX 1.1) [75].

One of the most difficult challenges in the design and deployment of DL models in embedded devices is the requirement of a set of tools specifically designed for the selected embedded hardware architecture. The following subsections enumerate the resources that NXP brings for AI application development:

- **ISP**: The software image signal processing (SoftISP) includes several functions that are executed on the GPU of the NXP^®^ i.MX 8 family device, i.e., bad pixel correction, white balance, histogram equalization, high-quality demosaicing and high-quality noise reduction (Figure 4). This pipelined image-processing engine, designed to take in high-speed video streams, is optimized for the on-chip GPU using two computing standards, OpenCL™ 1.2, and OpenVX™.

To take advantage of the ISP, the NXP partner Au-Zone [73] provides a highly optimized software libraries called Vision-Pack [74]. Vision AI Acceleration Library (VAAL) is a core library of Vision-Pack. On the input processing side VAAL offers accelerated image processing functions which connects cameras and codecs to image and graphics processors through DMA to the NPU or GPU. On the output processing side, provides accelerated decoding and interpretation of model results to minimize the time from inference results to user-readable data structures. These libraries have been used during the evaluation of the custom platform to optimally get the video from a camera to the inference engine and visualize the results.

**Figure 5:**
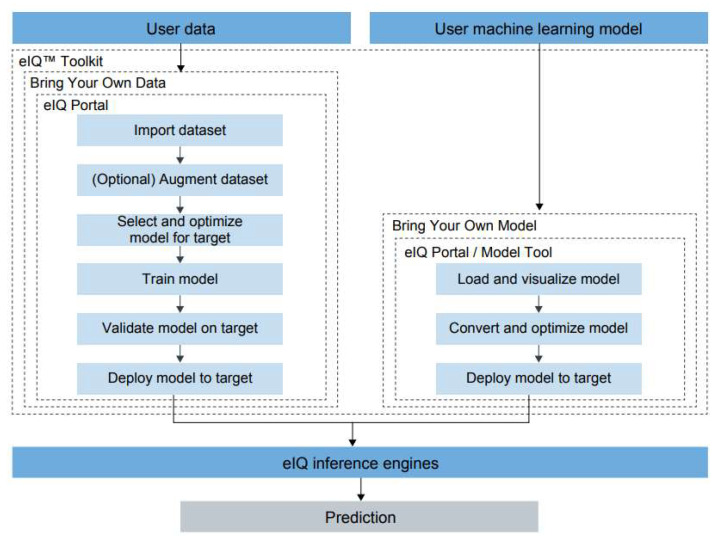
eIQ block diagram presenting the tools available to develop, analyze and deploy a custom model bringing your own data or bringing your own model [76].

**Figure 6:**
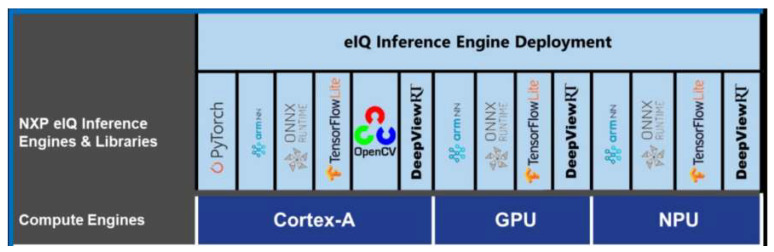
Inference Engines and libraries available for Neuronal Network Model Deployment for the NXP I.MX8M Plus platforms [77].

- **eIQ tools**: The *eIQ Toolkit* enables machine learning development through an intuitive GUI, *eIQ Portal,* and development workflow tools, along with command line host tool options that simplifies vision-based ML solutions development. It enables graph-level profiling capability with runtime insights to help optimize neural network architectures on target processors. The *eIQ Portal* provides two workflow pipelines according to user requirements. In the “bring your own data” (BYOD) pipeline, *eIQ Portal* takes the user data and generates a fully deployable DNN inference model using an adequate proprietary model zoo instance. In the “Bring your own model” (BYOM), an already existing model is modified and optimized to be compatible with one of the embedded AI run time libraries, as described below (Figure 5).

- **eIQ inference engines:** The *eIQ Tolkit* provides an output model that seamlessly feeds into the embedded run time. The available options are DeepViewRT, TensorFlow Lite, TensorFlow Lite Micro, Glow and ONNX runtime inference engines, as seen in the Figure 6. The *eIQ inference engine* library allows one to deploy the model into an appropriate hardware processor and to control the inference execution. In this work a model related with the NPU is tested and evaluated.

#### 2.2.2. Deep Learning Frameworks and Models

To test the performance of the custom AI Embedded Platform along with the accompanying inference engines, we have made use of models provided by Au-Zone and state of the art models provided by Model Zoo from Tensorflow.

An already trained SSD-MobilenetV2 networkwas downloaded from Tensorflow ModelZoo [78]. Then, the model was transformed to TensorFlow Lite using a Tensorflow script (model (1) in Table 1), and to a DeepViewRT format using the eIQToolkit (model (2) in Table 1).

In addition, already trained models provided by eIQTolkit and Au-Zone were considered, models (3–5). These models are specifically generated to be used in I.MX8M Plus processor with DeepViewRT inference engine.

The two embedded run times, TensorFlow Lite library and DeepviewRT have a Python and C++ API that allows the integration of deep learning models into embedded applications. This API is also responsible for converting and deploying the highly parallel tensor calculus into NPU to optimize both performance and processing time. DeepViewRT was developed by a NXP partner Au-Zone. In [79], there is a detailed description of the run time specifications. For each model architecture and model format, a slightly different inference Python script was developed.

### 2.3. Video Analytics Edge Computing (VAEC) System

The Video Analytics Edge Computing (VAEC) system developed by ICCS is documented in detail in [68]. The VAEC system is integrated with the DECIoT in order to provide enhanced situation awareness for person detection through a video streaming feed on an embedded edge device with GPU processing (via a NVIDIA Jetson RTX2) and a lightweight object detection deep learning scheme. In this study, additional experiments and a new training process for several altitudes and perspective views in terms of real-world scenes are conducted though VAEC system.

The VAEC system adopts a lightweight deep learning model with a CNN architecture for object detection, that is the pre-trained YOLOv5s [80], with high inference speed (70 ms) [68]. In the literature, YOLO in several versions has been considered as one of the most robust and efficient published deep learning based object detection frameworks [81,82,83,84]. The last version, YOLOv5, seems to have a great potential for object detection tasks in several applications with various challenges, such as complexity of the scene, light conditions, viewing perspective of the objects, etc. [85,86,87]. The YOLOv5s is trained by the well-known COCO dataset that contains 80 classes and more than 2,000,000 labeled images [88].

In [68], the performance of VAEC system was evaluated through several real-time experiments for person detection in the following terms: (i) in several light conditions, and (ii) using several types of camera sensors. However, the aforementioned experiments considered only the pre-trained YOLOv5s in: (i) outdoor cases, (ii) low-altitude camera views, and (iii) with one person presented in the scene. In this paper, we expand the evaluation of VAEC system to:

- Indoor cases associated with real-world applications (arson and burglary/space violation) from a free available dataset using the pre-trained YOLOv5s (see Section 3.2.1).
- Outdoor cases utilizing representative images from free available datasets: (i) of a variety of viewing perspectives, (ii) from high-altitude and unmanned aerial vehicle (UAV) camera views, and (iii) with complex city background. As mentioned above, the pre-trained YOLOv5s model has been trained mainly with terrestrial/low-altitude camera view RGB imagery from the COCO dataset. Thus, to cover cases of high-altitude and UAV camera views and the associated viewing perspectives, a custom deep learning model is created (see Section 3.2.2).

The Python programming language (version 3.6) and the libraries TensorFlow [89] and OpenCV [90] were mainly used for the object detection processes and the re-training process.

For the quantitative assessment of the person detection process, four objective criteria were adopted according to the International Society for Photogrammetry and Remote Sensing (ISPRS) guidelines [91,92], namely completeness (C_M_), correctness (C_R_), quality (Q), and F1 score measures per object (person), given as:

(1) $$C_{M}=\frac{\left\|TP\right\|}{\left\|TP\right\|+\left\|FN\right\|},C_{R}=\frac{\left\|TP\right\|}{\left\|TP\right\|+\left\|FP\right\|},Q=\frac{\left\|TP\right\|}{\left\|TP\right\|+\left\|FP\right\|+\left\|FN\right\|},F1=2\times \frac{C_{R}\times C_{M}}{C_{R}+C_{M}}$$

where TP, FP, and FN denote true positives, false positives, and false negatives, respectively. The TP entries are the persons that exist in the scene and were, thus, correctly detected. The FP entries are the persons that do not exist in the scene and were, thus incorrectly detected (assigned as false detected persons). The FN entries are the persons that exist in the scene but were not detected (assigned as missed persons).

## 3. Results

In this section, the results obtained from the custom AI embedded platform and the VAEC system via extended experiments are presented. Concerning the custom AI embedded system, the combination of hardware specific neuronal network and image processing tools and the multi-threading approach has resulted in a reduction of the whole processing time, allowing a theoretical rate of 60 FPS or around 15 ms of total computing time. Concerning the VAEC system, the achieved results for both the pre-trained and custom YOLOv5s are considered to be satisfactory, proving their suitability and efficiency for real-word applications in smart cities. More details concerning the experiments and the relevant results are provided in the following sub-sections.

### 3.1. Custom AI Embedded Platform Experiments

Low-cost embedded devices are restricted by the processing capabilities endowed on it. It is, therefore, essential to make use of its resources in the most effective form. In previous work [93], the custom AI Embedded Platform developed by Tekniker in the context S4allCities project was presented. This platform has been developed to provide low cost and low consumption edge deep learning capabilities. The novelty in this case is set on the hardware specific tools available to accelerate the application runtime rather than on evaluating a specific model performance. This work evaluates the execution time reduction obtained by Tensorflow Lite and the DeepViewRT inference tools provided by eIQ Framework, as well as by the VisionPack to perform image acquisition operations.

**Figure 7:**
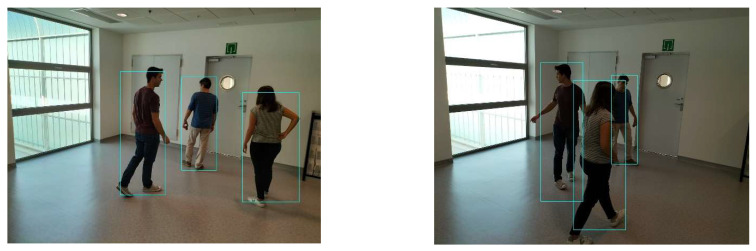
Setup scenario and person detection results obtained with the I.MX8M Plus-based embedded board. **Left**, non-overlapped persons detected. **Right**, overlapped persons detected.

The implemented application consists of monitoring a human restricted area and sending alerts to the SmartCity middleware with the number of people in the image and video streaming in case of human presence. This information is transmitted via MQTT to the DECIoT and from there resent via Apache Kafka where the high-level smart city operators evaluate if the people on the image have authorization or not. For testing purposes, a restricted area has been simulated at Tekniker facilities. The result of the detection inference is shown in Figure 7 below. 

The subtasks that need to be performed are image acquisition, preprocessing and scaling, detection inference and results publications. The results consist of a JSON message containing an event with the number of people in the restricted area and an image video streaming directly to the smart city through the RTP protocol. The software status in previous work [93] required long processing times for image acquisition and inference of a simple deep learning model. The obtained times at this stage were 4 ms for scaling the image to be suitable for the DL model, 15 ms for the inference of an image and, finally, 0.5 ms for both publishing the event as JSON message and streaming the image. The script uses a highly optimized thread architecture to parallelize as much as possible the various tasks defined above leading to an image processing rate of 20 FPS. Those preliminary experiments used the model numbered as 1 in Table 2.

In order to enhance the efficiency and reduce the processing time of this and future deep learning vision applications, some experiments were carried out using optimized libraries for model quantization and model deployment. In particular, Au-Zone Vision-Pack for improved image preprocessing and buffering operations and DeepViewRT along with eIQ Toolkit in the host for model inference optimization were used.

Due to the different model formats and libraires used in the experiments, it was necessary to implement some application scripts in addition to the original one. A brief description of each one is shown in Table 2.

The experiments carried out are summarized in Table 3. As shown, the experiments use a mixed combination of script, model and processor, leading to a quite heterogeneous time results.

The first conclusion derived from Table 3 is that there is a huge improvement when the NPU is used compared with the CPU. The NPU is around 16 times faster than CPU. In addition, the use of the NPU frees the CPU to be used for another tasks, so the inference of the model can be paralleled with the camera image acquisition to achieve higher performances.

Image preprocessing has also been highly improved, reducing the image scaling time by a factor of 4.5 in the last experiments. This has been achieved with the use of the Vision-Pack library resources. The main difference is that the first experiments use a OpenCV function executed by CPU, while the last experiments use an optimized video pipeline with internal GPU image processing and DMA memory operations. Again, the CPU is freed and is, therefore, available to attend other tasks.

Finally, the inference time was also improved. The experiments performed with the highly optimized models (4–5) provided by Au-Zone and the execution using the VAAL library, leads to a factor of 2 reduction in the inference time compared with the original model (1).

### 3.2. Video Analytics Edge Computing (VAEC) System Experiments

#### 3.2.1. Experiments with the Pre-Trained YOLOv5s

This study is focused on the person detection; thus, only the class “person” of the pre-trained YOLOv5s weights was activated. The experiments were conducted by utilizing some representative videos from the public dataset of UCF-Crime [94] associated with arson and burglary/space violation activities. The probability percentage threshold associated with the detected person was selected as 20%.

Table 4 depicts representative consecutive video frames for five selected videos (V1 to V5) of the UCF-Crime dataset associated with the person detection results (red bounding boxes and the relevant detection probability percentages superimposed to the video frames). The quantitative assessment results for the person detection process for videos V1 to V5 are provided in Table 5. The achieved results of the pre-trained YOLOv5s (average values) were C_M_ = 81.2%, C_R_ = 90.2%, Q = 74.9%, and F1 = 85.3%.

#### 3.2.2. Experiments with the Custom YOLOv5s

The YOLOv5s is retrained applying a transfer learning scheme [95] utilizing only one class (“Person”) and exploiting the available pre-trained YOLOv5s weights. To this end, several free available video/image datasets were selected, in which the whole human body pose (and not parts of persons or occluded persons) of moving or standing persons is depicted and annotated from high-altitude and UAV camera views. The selected datasets were (i) OKUTAMA [96], (ii) VisDrone2019 [97], (iii) P-DESTRE [98], (iv) AU-AIR [99], (v) IRICRA [100] and (vi) OTCBVS-THERMAL [101].

Table 6 depicts sample views from each dataset, the type of the used camera sensor (i.e., RGB or thermal), as well as the corresponding number of images and the number of the samples of the class “Person”. The total count of the samples indicates an adequate training and validation set depicting a variety of scenes captured from several spectral sensors and perspectives, and with variable pixel resolution. Thus, a robust custom deep learning model is able to be extracted with high generalization properties in videos and images from the training set. Each dataset is split into a training set and validation set. The corresponding percentages of the total images of each dataset are 80% for the training set and 20% for the validation set. In addition, the corresponding annotation files are created both for the training and validation set. Concerning the training process: (i) the batch size was selected to be equal to 120, (ii) the learning rate was selected to be equal to 0.01, and (iii) a GPU NVIDIA GeForce RTX 3090 was utilized. The total number of the training epochs was 80, while the total computational time of the training process was 3 d 19 h.

**Figure 8:**
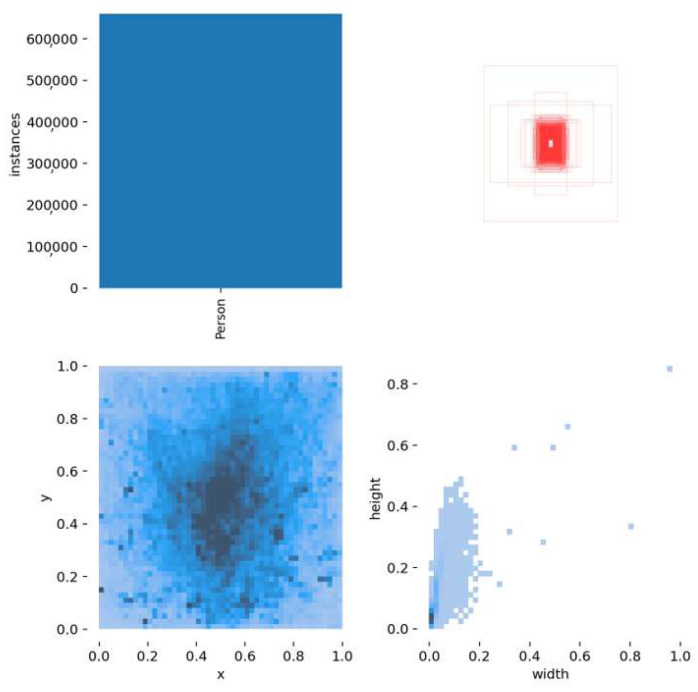
Details of the total dataset. **Top left** indicates that the dataset has only one class, i.e., Person. **Top right** shows the shapes of the bounding boxes, as well as their orientation. **Bottom left** depicts the location of the center of each bounding box in the total dataset images in which darker pixels imply that more bounding boxes in these areas exist. **Bottom right** shows the width and height of the bounding boxes.

In Figure 8, some details of the total dataset are provided. The top left indicates that the dataset has only one class, i.e., “Person”. Top right shows the shapes of the bounding boxes, as well as their orientation. The bottom left depicts the location of the center of each bounding box in the total dataset images, in which darker pixels imply that more bounding boxes exist in these areas. Finally, bottom right shows the width and height of the bounding boxes; since the custom model is trained from videos/images captured from high-altitude and UAV camera views, the bounding boxes of the depicted persons in them are quite small.

**Figure 9:**
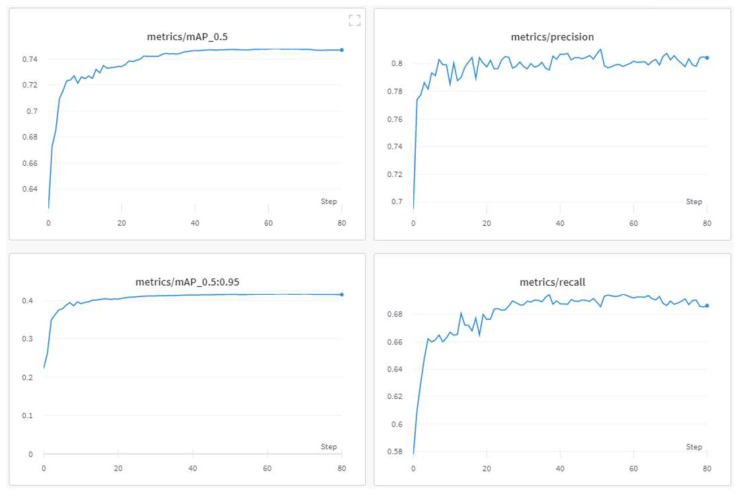
Basic training metrics of the custom model.

**Figure 10:**
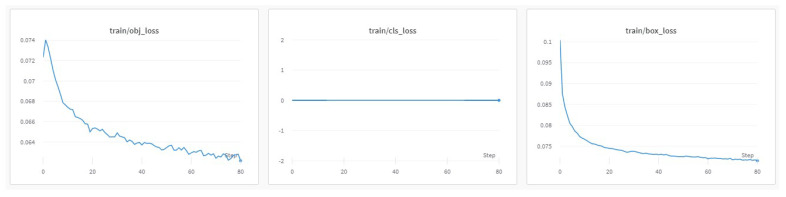
Training set metrics.

**Figure 11:**
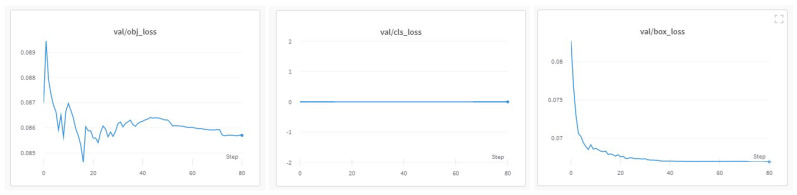
Validation set metrics.

The progress of the training process during the 80 epochs is shown in Figure 9, Figure 10 and Figure 11. The basic training metrics of the custom model are the following. (i) “Box loss” that represents how well the algorithm can locate the center of an object and how well the predicted bounding box covers an object. (ii) “Obj loss” that measures the difference of the predicted “objectness” with the ground truth “objectness”; “objectness” is essentially a measure of the probability that an object exists in a proposed region of interest. If the objectivity is high, this means that the image window is likely to contain an object. (iii) “Cls loss” or (“Class loss”) that measures how well the algorithm can predict the correct class of a given object (if class loss is absolute zero, it means the model is trained to detect the objects only and not classify them). (iv) “Precision” that measures how accurate (percentage) are the model’s predictions. (v) “Recall” that measures how good the model finds all the positives; for example, the model can find 69% of the possible positive cases in top-k predictions. (vi) The mean average precision (mAP).

In Figure 9, during the first 25 epochs, all metrics converged on a constant value. What can be derived from the precision and recall graphs is that false positive and false negative detections did not decrease and oscillated around 81% and 69%, respectively, after the 25th epoch. Furthermore, both mAPs implied the same conclusion, as well as that the overall performance did not improve. It is noted that after the 20th epoch, there was no significant progress to the model’s performance. The training losses decreased with progressive epochs; however, what matters is the validation losses and most of all the mAPs.

In Figure 10, in the training losses, class loss was zero since no object classification was applied. The progress of the training “objectness” loss showed that the model detected objects from the start of training, reached a peak for the first epochs, and converged at the 80th epoch. The training box loss progression implies that as more epochs passed, the model performed well on the training dataset. In the validation losses (Figure 11), class loss was zero as well. In contrast with the training “objectness” loss, the validation “objectness” loss fell very quickly during the first few epochs. However, there was no major improvement during the next epochs. The same applied for the validation box loss.

To evaluate the custom model in real world scenes, additional datasets were utilized that were not used in the training and validation processes. Such datasets are (i) MINI-DRONE [102], (ii) CROWD HUMAN [103], (iii) FDST [104] and NWPU [105]. The probability percentage threshold associated with the detected person was selected as 50%. Table 7 depicts representative video frames and images of the aforementioned additional dataset associated with the person detection results (red bounding boxes and the relevant detection probability percentages superimposed to the video frames and images). The relevant quantitative assessment results are provided in Table 8. The achieved results (average values) of the custom YOLOv5s were C_M_ = 85.3%, C_R_ = 96.4%, Q = 82.7%, and F1 = 90.4%.

Additionally, a comparison between the custom YOLOv5s with other studies that adopt YOLOv5 or other detectors was carried out, focused on in person detection from high-altitude and UAV camera views (Table 9). According to this comparison, all the metrics of the custom YOLOv5s are high, indicating its efficiency.

## 4. Discussion and Conclusions

The objective of this work is to highlight the utility and functionality of the two proposed different schemes with edge computing capabilities in smart city applications focused on person detection: (i) the custom AI Embedded Platform based on the I.MX8 PLUS NXP processor, and (ii) the VAEC system that adopts a pre-trained and a custom YOLOv5s deep learning model.

The experiments on the custom AI Embedded Platform have demonstrated the competence of the device for a person detection use case and the improvements obtained by the use of NXP specific tools. The improvements obtained are as follows. (i) Inference reduction through quantization of the network, conversion to .rtm format and deployment on the NPU. However, the conversion cannot start from TFLite directly as the box decoding portion of the model, when quantized, cannot be embedded into the DeepViewRT model due to some missing parameters. When converting from the full SavedModel, the DeepView converter is able to retrieve all required parameters to generate a fully quantized model [111]. The obtained improvements go from 15 ms to 7 ms. (ii) Additionally, the image scale processing time has been remarkably reduced by a factor of 4.5 from 4 ms to 0.89 ms. The VAAL library manages the vision pipeline through the use of GPU and Direct Memory Access buffers.

**Figure 12:**
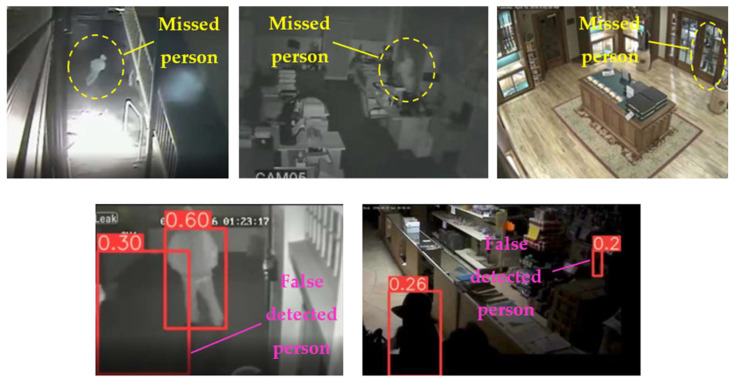
Observed FN entries (yellow ellipse with dashed line) and FP entries (magenta indications) during the person detection process applying the pre-trained YOLOv5s.

In the case of the VAEC system, the achieved results of the pre-trained YOLOv5s are considered to be satisfactory and are quite similar to those of [68]. This indicates a homogeneous and stable performance of the pre-trained YOLOv5s both in indoor and outdoor environments. The observed FN entries were mainly due to partial/total occlusions or to a local failure of the YOLOv5s (i.e., persons that were unrecognizable by the algorithm). Such FN entries led to missed persons and, therefore, to the reduction of the C_M_ rate. On the other hand, the observed FP entries were mainly due to: (i) artificial objects’ patterns that were similar with those of persons, and (ii) similar spectral values between objects in the background scene and the persons. Such FP entries led to the reduction in the C_R_ rate. Figure 12 shows representative examples of FP and FN entries by applying the pre-trained YOLOv5s.

**Figure 13:**
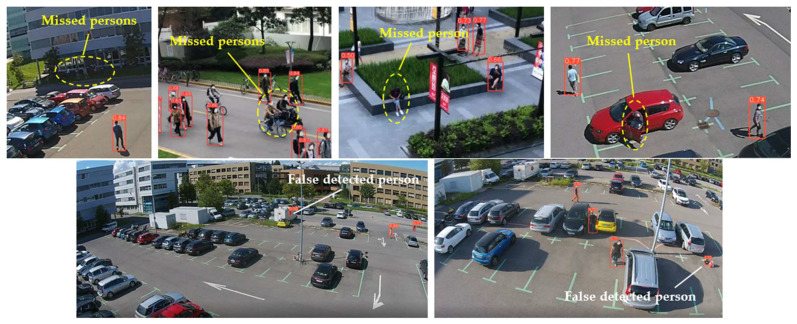
Observed FN entries (yellow ellipse with dashed line) and FP entries (white indications) during the person detection process applying the custom YOLOv5s.

On the other hand, considering the complexity of the background scene, as well as the person’s size variation and perspective view from high-altitude and UAV camera positions, the results of the custom YOLOv5s are satisfactory. The observed FP entries were mainly due to artificial objects’ patterns that were similar with those of persons. Since the person’s size in the annotated videos/images used for the training process is quite small, it is quite possible for the algorithm to fail in such cases, especially in very complex scenes. However, according to Table 8, a high average C_R_ rate (larger than 95%) was achieved, indicating few FP entries. On the other hand, the average C_M_ rate was smaller than the average C_R_ rate due to most observed FN entries. Such FN entries led to missed persons due to: (i) partial/total occlusions of persons (between them in crowed areas or by other objects); (ii) the human body pose of the persons was not similar with the one considered in the training process, e.g., seated persons were not detected since only moving or standing persons were mainly annotated according to the available datasets; and (iii) similar spectral values between objects in the background scene and the persons, e.g., in shadowed areas. Figure 13 shows representative examples of FP and FN entries by applying the custom YOLOv5s.

Several open-source edge computing frameworks have gained significant attention in the last years due to their open use, efficiency and flexibility. According to this research, the EdgeX Foundry was considered as the most suitable edge computing framework for this study. Thus, the designed and developed Distributed Edge Computing IoT Platform (DECIoT) was based on the EdgeX Foundry. The DECIoT platform addresses, among others, the problem of gathering, filtering and aggregating data, allows the interoperability between devices, interacts with the IoT devices, provides security and system management, provides alerts and notifications, executes commands, stores data temporarily for local persistence, transforms/process data and, in the end, exports the data in formats and structures that meet the needs of other platforms. The approach described in this paper demonstrates the suitability of edge computing systems for machine vision applications for safety and security in smart cities. These devices can be widely spread to monitor the city, detect anomalies or different human behaviors, and automatically generate events that are evaluated in higher contexts by human operators. This approach was demonstrated through two different edge computing systems developed within the framework of the S4AllCities EU funded project. The aim of this work was not to compare both systems but to focus on different metrics, as highlighted below.

On the one hand, a low-cost edge inspection vision system was developed based on the i.MX8M and its Neural Co-Processing Unit (NPU). This platform makes use of novel hardware specific tools from NXP, such as the DeepViewRT model inferencing framework and the Image Signal Processing (ISP), to reduce the processing time of a person detector application presented in [93]. The actions taken to optimize the application are as follows. (i) Accelerating the deployment of the model and its inference by a factor of 16. This is achieved by the use of DeepView converted models and execution of those through the VAAL library. (ii) The VAAL library was also used to reduce the time of resizing an image by a factor of 4.5. (iii) The entire people detection task was divided into smaller tasks and processed in individual threads. The combination of hardware specific neuronal network and image processing tools and the multi-threading approach resulted in a reduction of the whole processing time, allowing a theoretical rate of 60 FPS or around 15 ms of total computing time.

On the other hand, a commercial NVIDIA Jetson RTX2 was used to evaluate a pre-trained and a custom YOLOv5s deep learning model. The achieved results of both the pre-trained and custom YOLOv5s adopted from the Video Analytics Edge Computing (VAEC) system are considered to be satisfactory, proving their suitability and efficiency for real-word applications in smart cities. The average Q and F1 metrics of the custom YOLOv5s (Q = 82.7% and F1 = 90.4%) are better than the ones of the pre-trained YOLOv5s (Q = 74.9% and F1 = 85.3%). Additionally, both in the pre-trained and custom YOLOv5s, the average C_R_ is larger than the C_M_ rate by about 10%. This means that both models are precise and few FP entries are observed during their utilization. Although the average C_M_ rate of both models is larger than 80%, several FN entries were observed. The observed FN and FP entries were observed mainly due to: (i) partial/total occlusions of persons (between them in crowed areas or by other objects), (ii) local failure of the algorithm, (iii) artificial objects’ patterns that were similar with those of persons, (iv) similar spectral values between objects in the background scene and the persons (e.g., in shadowed areas), and (v) the human body pose of the persons was not similar with the one considered in the training process (e.g., in the custom model, some seated persons were not detected since only moving or standing persons were mainly annotated according to the available datasets). However, the custom YOLOv5s focused on high-altitude views while the pre-trained YOLOv5s focused on low-altitude views. Thus, it can be considered that they can be complementary, depending on the use case in a smart city application, e.g., the custom model can be exploited under a UAV on-board processing, while the pre-trained can be exploited under terrestrial processing. In any case, both models achieved an effective and stable performance in indoor and outdoor environments.

## Figures and Tables

**Table 1:** Models used for inference tests of the Tekniker AI Embedded Platform.

#	Model Name	Format	Description
1	mobilenet_ssd_v2_coco_quant_postprocess	tflite	Model converted from Tensorflow format to tflite using a Tensorflow script
2	mobilenet_ssd_v2_coco_quant_postprocess	rtm	The model above converted to DeepViewRT using eIQTolkit
3	mobilenet_ssd_v1_1.00_trimmed_quant_anchors	rtm	A proprietary model distributed with eIQTolkit
4	mobilenet_ssd_v2	rtm	A model provided by Au-Zone obtained from available Model Zoo 1 model and converted to .rtm
5	modelpack_people_320 × 320	rtm	A model provided by Au-Zone obtained from available Model Zoo 2 model and converted to .rtm

**Table 2:** Brief explanation of the Python scripts used for testing purposes.

#	Script Name	Description
1	video_stream_tflite.py	Python script executing a .tflite model.
2	video_stream_rtm.py	Python script executing a .rtm model with DeepViewRT engine.
3	video_ stream_rtm_VisionPack.py	Python script executing a .rtm model with DeepViewRT engine and additional VAAL library.

**Table 3:** Experiments results Summary. All times are in milliseconds.

#	Model Used	Script Used	Inference Processor	FPS ^(1)^	Image-Scaling (ms)	Inference (ms)	Publish Results (ms)
1	1	1	CPU	30	4	254	0.5
2	1	1	NPU	30	4	15	0.5
3	2–3	2	CPU	30	4	280	0.5
4	2–3	2	NPU	30	4	33	0.5
5	4	3	NPU	30	0.89	8–9	0.5
6	5	3	NPU	30	0.89	7	0.5

^(1)^: The frame rate (FPS) is defined by the camera driver configuration. The OV5640 camera used has a maximum rate of 30 FPS.

**Table 4:** Qualitative person detection results per selected video via the pre-trained YOLOv5s.

Dataset Video ID			
V1	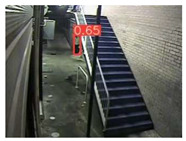	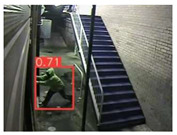	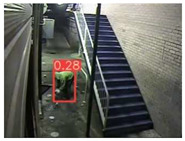
	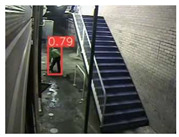	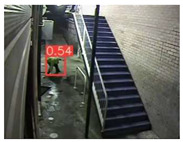	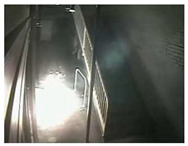
V2	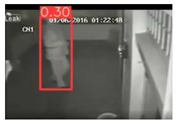	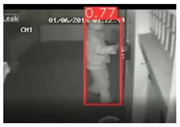	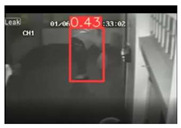
	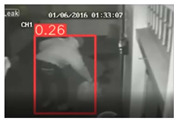	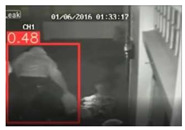	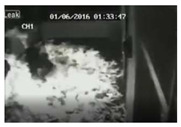
V3	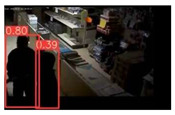	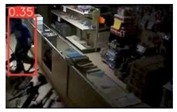	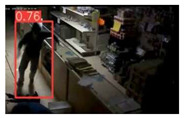
V4	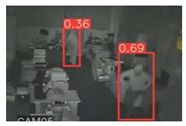	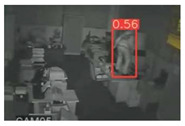	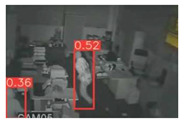
V5	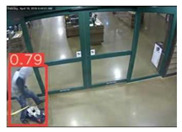	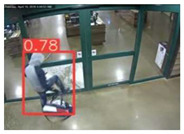	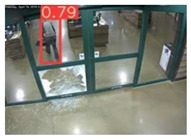
	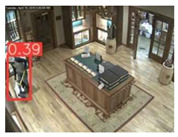	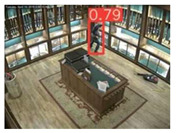	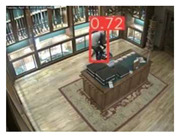

**Table 5:** Quantitative person detection results per selected video via the pre-trained YOLOv5s.

Dataset Video ID	C_M_ (%)	C_R_ (%)	Q (%)	F1
V1	83.3	100.0	83.3	90.9
V2	80.0	94.1	76.2	86.5
V3	81.8	81.8	69.2	81.8
V4	75.0	75.0	60.0	75.0
V5	85.7	100.0	85.7	92.3
Average	81.2	90.2	74.9	85.3

**Table 6:** Views from each selected dataset for the training process.

Dataset	Type of Sensor	View		Number of Images	Number of Samples of the Class “Person”
OKUTAMA	RGB	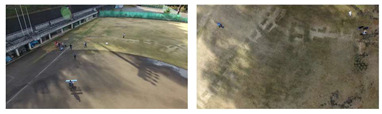		4128	23,749
VisDrone2019	RGB	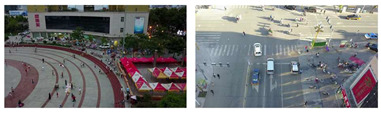		34,061	406,896
P-DESTRE	RGB	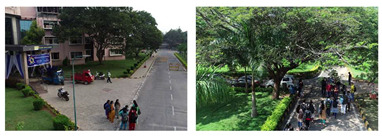		20,635	292,003
AU-AIR	RGB	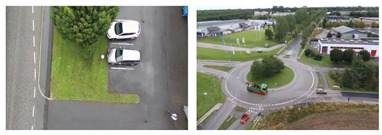		32,713	5158
IRICRA	Thermal	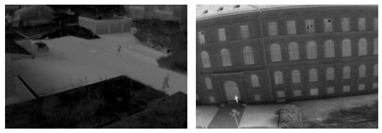		3237	5747
OTCBVS-THERMAL	Thermal	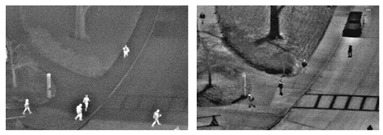		659	2034
			Total count	95,433	735,587

**Table 7:** Qualitative person detection results per selected videos/images via the custom YOLOv5s.

Dataset	View	
MINI-DRONE	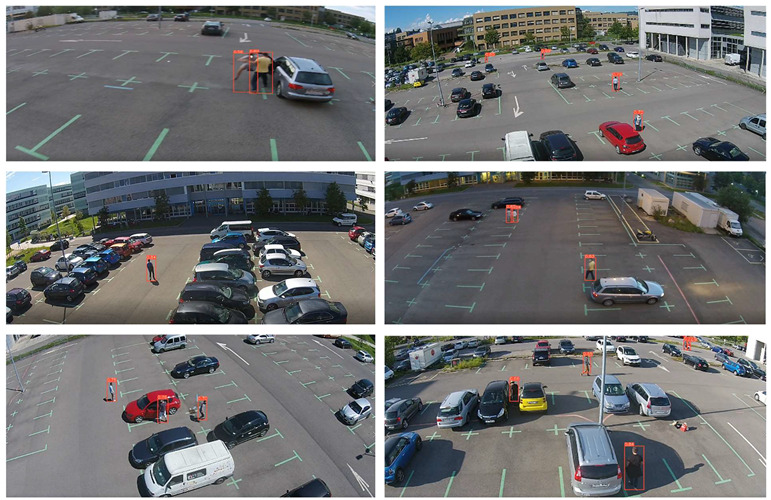	
CROWD HUMAN		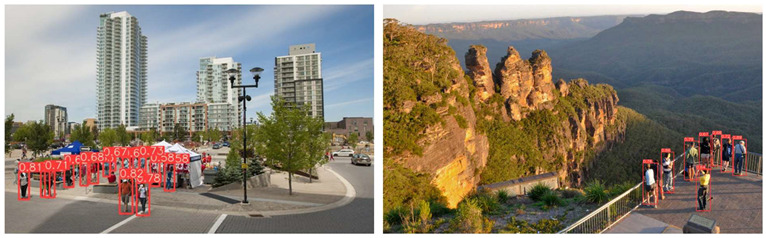
FDST		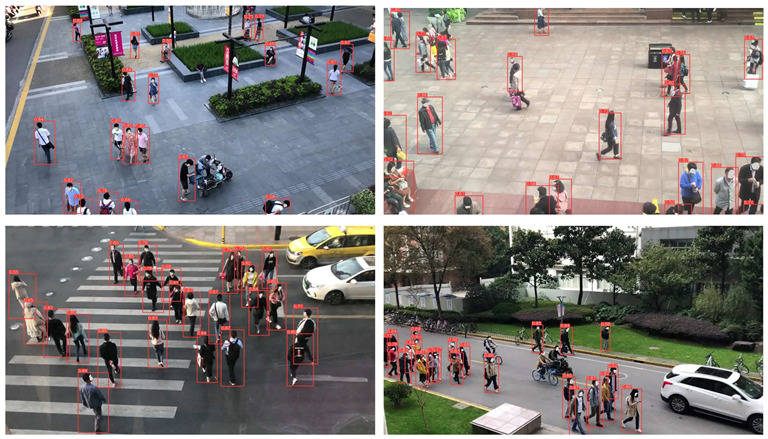
NWPU		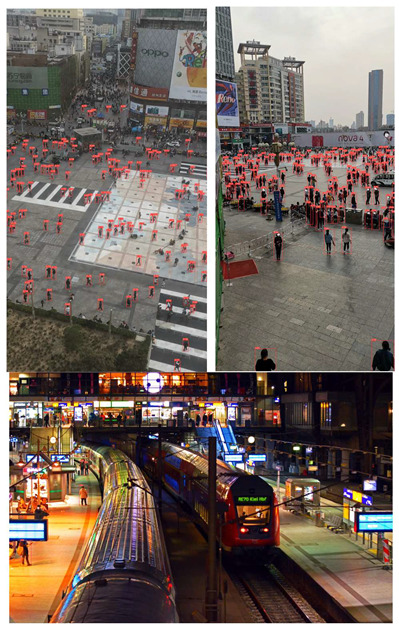

**Table 8:** Quantitative person detection results per selected videos/images via the custom YOLOv5s.

Dataset	C_M_ (%)	C_R_ (%)	Q (%)	F1
MINI-DRONE	85.3	85.5	74.5	85.4
CROWD HUMAN	92.3	100.0	92.3	96.0
FDST	84.1	100.0	84.1	91.4
NWPU	79.7	100.0	79.7	88.7
Average	85.3	96.4	82.7	90.4

**Table 9:** Comparison between the custom YOLOv5s with other studies that adopt YOLOv5 or other detectors focused in person detection from high-altitude and UAV camera views.

Method	Dataset	C_M_ (%)	C_R_ (%)	Q (%)	F1
Yolov5 [106]	VisDrone2022 [107]	83.7	79.8	69.1	81.7
Yolov5 + SGB [108]	OKUTAMA	75.4	67.4	55.3	71.0
SSD + CNN [109]	OKUTAMA	28.3	-	-	-
Improved Yolov5 [110]	VisDrone2022 [107]	97.1	84.3	82.2	90.2
custom YOLOv5s	MINI-DRONE CROWD HUMAN FDST NWPU	85.3	96.4	82.7	90.4

## Data Availability

Data sharing is not applicable to this article. The following open available datasets were used within this work: COCO [88], UCF-Crime [94], OKUTAMA [96], VisDrone2019 [97], P-DESTRE [98], AU-AIR [99], IRICRA [100], OTCBVS-THERMAL [101], MINI-DRONE [102], CROWD HUMAN [103], FDST [104] and NWPU [105].
